# Design and Experimental Validation for Direct-Drive Fault-Tolerant Permanent-Magnet Vernier Machines

**DOI:** 10.1155/2014/241085

**Published:** 2014-06-17

**Authors:** Guohai Liu, Junqin Yang, Ming Chen, Qian Chen

**Affiliations:** School of Electrical and Information Engineering, Jiangsu University, Zhenjiang 212013, China

## Abstract

A fault-tolerant permanent-magnet vernier (FT-PMV) machine is designed for direct-drive applications, incorporating the merits of high torque density and high reliability. Based on the so-called magnetic gearing effect, PMV machines have the ability of high torque density by introducing the flux-modulation poles (FMPs). This paper investigates the fault-tolerant characteristic of PMV machines and provides a design method, which is able to not only meet the fault-tolerant requirements but also keep the ability of high torque density. The operation principle of the proposed machine has been analyzed. The design process and optimization are presented specifically, such as the combination of slots and poles, the winding distribution, and the dimensions of PMs and teeth. By using the time-stepping finite element method (TS-FEM), the machine performances are evaluated. Finally, the FT-PMV machine is manufactured, and the experimental results are presented to validate the theoretical analysis.

## 1. Introduction

Currently, the permanent-magnet vernier (PMV) machine is becoming a promising candidate for direct-drive application such as the electric vehicle propulsion and the wind power generation [1–4]. For conventional direct-drive motor topologies, magnetic circuits with a large number of poles, slots, and windings are usually required to generate high torque at low speed. Such machine design will inevitably increase the machine volume and weight. However, it has been verified that a PMV machine with concentrated windings can offer high torque capability at low speed while reducing the number of slots and thus copper loss [1, 3, 4]. Based on the “magnetic gearing effect” [5, 6], PMV machines can effectively modulate the high-speed rotating field of the armature windings and the low-speed rotating field of the PM outer rotor by adopting the flux-modulation poles (FMPs). Hence, the armature poles number can differ from the rotor poles one, which breaks the traditional design rule of PM machines. Also, the volume of the stator with vernier structure will not increase with the large number of PM poles.

On the other hand, besides the requirements of high torque density, high reliability operation of motor drives is very important in such direct-drive applications [7, 8]. Thus, the fault-tolerant capability of motor drives is becoming attractive. A number of researchers have investigated various motor topologies for fault-tolerant applications. Due to its simple and rugged construction, the switched reluctance motor has the advantage of fault-tolerant capability [9, 10]. However, it still suffers from the drawback of relatively low power density. A PM machine is chosen in preference to switched reluctance motor due to its high power density, high efficiency, and equal fault-tolerance [11]. The key to fault tolerance in PM machines is to have both a sufficiently high phase inductance to limit short circuit currents and a sufficiently low mutual inductance between phases to avoid the performance degradation in the remaining healthy phases. Design methods of special slot-pole combination and concentrated windings can offer this characteristic as demonstrated in [11]. In addition, the increase of the phase number is also an effective way to improve reliability in motor drives. Actually, fault-tolerant feature is a significant advantage of multiphase machines over conventional three-phase machines [12]. For example, in a five-phase PM machine, the loss of up to three phases will not prevent the machine from continuous operation or even startup.

In this paper, a five-phase fault-tolerant PMV (FT-PMV) machine will be analyzed and designed, aiming to incorporate the merits of the PMV machine and the fault-tolerant machine. The operation principle of the proposed machine will be presented. The design process and optimization are presented specifically, such as the combination of slots and poles, the winding distribution, and the dimensions of PMs and teeth. By using the time-stepping finite element method (TS-FEM), the machine performances will be assessed. Finally, experimental results will be provided to validate the theoretical analysis.

## 2. Principle of Operation

The key of a PMV machine is the introduction of FMPs. Its function is to modulate the high-speed rotating field generated by armature windings to the low-speed rotating field in the air-gap. This low-speed rotating air-gap field in turn interacts with the PM poles surface-mounted on the outer rotor, thus creating the desired low-speed high-torque operation. Since this operating principle is based on the “magnetic gearing effect,” the relationship among the number of PMs pole pairs, armature winding fundamental pole pairs, and FMPs should be satisfied.

The number of pole pairs in the space harmonics of the flux density distribution and the corresponding rotational speed can be expressed as

(1) $$\begin{array}{c} p_{i,m,k}=\left|mp_{i}+kn_{s}\right|,\\ \omega_{i,m,k}=\frac{mp_{i}\omega_{i}}{\left|mp_{i}+kn_{s}\right|}, \end{array}$$

where *m* = 1, 3, 5,… (according to all the PMs with different pole pairs and most concentrated windings) and *k* = 0, ± 1, ± 2,…, *i* = 1, 2, and *i* = 1 implies that the harmonic component is excited by the armature windings, while *i* = 2 means that it is excited by the outer rotor PMs, *n*_*s*_ is the number of the FMPs, and *ω*_1_ and *ω*_2_ are the rotational speeds of the armature fundamental field and outer rotor, respectively. For a clear discussion, it is assumed that the harmonic component is excited by the armature windings; namely, *i* = 1. Meanwhile, the maximum harmonic component only can be obtained based on *k* = ±1 after modulation. So, by selecting *k* = −1 (1) can be rewritten as

(2) $$\begin{array}{c} p_{1,m,-1}=n_{s}-mp_{1}, \end{array}$$

(3) $$\begin{array}{c} \omega_{1,m,-1}=\frac{mp_{1}\omega_{1}}{n_{s}-mp_{1}}. \end{array}$$

In order to transmit torque at different speeds, the pole pairs of the outer PMs (*p*_2_) must be equal to *p*_1,*m*,−1_; namely, *p*_2_ = *n*_*s*_ − *mp*_1_, *ω*_2_ = *mp*_1_ *ω*_1_/*n*_*s*_ − *mp*_1_. Thus, the high-to-low speed ratio *G*_*r*_ is governed from (3) by

(4) $$\begin{array}{c} G_{r}=\frac{\omega_{1}}{\omega_{2}}=\frac{n_{s}-mp_{1}}{mp_{1}}. \end{array}$$

Since *m* is 1, 3, 5,… (produced by Fourier decomposition), it is very necessary to get the optimal value of *mp*_1_ which means the order of the maximum magnetic field component induced by armature windings before modulation. When this *mp*_1_ is determined, the value of *p*_1,*m*,−1_ (*n*_*s*_ − *mp*_1_) can be easily obtained which means the order of the maximum magnetic field component induced by armature windings after modulation. As a result, only the PMs with *p*_2_ pole-poles (*p*_2_ = *p*_1,*m*,−1_) interact with this biggest *p*_1,*m*,−1_th space harmonic, and the machine can produce the maximum torque.

Usually, in conventional PM machines [13], the fundamental stator space harmonic *p*_1_th is the biggest; namely, *m* = 1. Actually, there is a traditional design rule that the number of pole pairs of fundamental stator space harmonic *p*_1_ and the number of rotor PM pole pairs *p*_*r*_ must be the same (*p*_1_ = *p*_*r*_). However, in the PM machines with fractional-slot concentrated windings (FSCWs) [14], this rule is not very necessary. The number of pole pairs of the fundamental stator space harmonic *p*_1_ may be smaller than that of the PM rotor *p*_*r*_. The torque is developed by the interaction of a higher order stator space harmonic with the PMs, which means *m* ≠ 1. Only if the *mp*th space harmonic interacts with the *p*_*r*_ pole pairs PM rotor, the machine can produce maximum continuous useful torque, which means *mp*_1_ = *p*_*r*_. As a result, to determine the value of *p*_2_ (*p*_1,*m*,−1_th), a PMV machine should have found its “original PM machine” with *p*_*r*_ pole pairs PMs, in which their armature windings are completely identical. This is due to *mp*_1_ = *p*_*r*_ = *n*_*s*_ − *p*_2_. To be concluded, various armature winding connections decide various vernier structures. The winding design of the proposed PMV machine will be presented in Section 3.2.

## 3. Machine Design and Optimization

First of all, the design objectives of the machine need to be clarified. The conventional design criteria are listed in Table 1. The outside stator radius *R*_1_, machine length *L*_*s*_, number of phases *N*_ph_, current density *J*_*a*_, and slot packing factor *k*_*s*_ are kept invariant. Since naturally cooled condition is chosen, the current density cannot be very high. Also, both high torque density and high reliability are required. According to the operation principle of PMV machines discussed in Section 2, the aim of high torque density can be achieved by adopting the vernier structure. In this section, the fault-tolerant design aimed to have high reliability will be detailed, while the vernier effect is still satisfied.

### 3.1. Main Dimensions

The initial design of any PM machine includes the determination of the outside stator radius *R*_1_, the outside rotor radius *R*_2_, the stack length *L*_*s*_, and the air-gap length *R*_*g*_. For a machine with specific flux density, electric and magnetic loading, and speed, its rated output power is proportional to the rotor volume. Usually, the air-gap length in PM machines has little impact on power since the permeance of PM is close to that of air. Hence, the thickness of PM is the dominant part in the flux path. However, for PMV machines, the harmonics in air-gap are used to produce torque, which means that PMV machines are very sensitive to the air-gap length. Meanwhile, compared with PM machines, PMV machines can have the thinner thickness of rotor and stator yoke due to their shorter flux path and larger pole number. Thus, the volume can be minimized. All these four parameters have been determined as a list in Table 3. The influences of the air-gap length, thickness of PM, and other critical parameters will be analyzed and discussed.

### 3.2. Design of Stator, Phases, and Winding Configuration

Since fault tolerance and high reliability are the foundations of this design, the minimum electrical, magnetic, and thermal interactions are required between phases. As a result, a fault in one phase does not undesirably affect the remaining healthy phases. These requirements can be satisfied by designing the appropriate armature windings with one-phase winding per slot. Meanwhile, this topology has a coil wound around a single tooth which is called single-layer FSCW (SL-FSCWs). This solution enhances thermal isolation and minimizes mutual coupling between phases. In addition, overlaps between end-windings of different phases are eliminated, so that the volume of copper can be significantly reduced, in particular when the axial length of the machine is small. Considering that any PMV machine can be transformed by its “original PM machine” discussed in Section 2, an “original PM machine” can be designed firstly before introducing the vernier structure.

In any SL-FSCW PM machine, the basic winding module (with the smallest allowable number of slots and poles) has just two antiphase coils per phase. For any such winding with *N*_ph_ phases and *S* slots, the required number of PM pole pairs *p*_*r*_ is given by [8]

(5) $$\begin{array}{c} 2p_{r}=S\left(1\pm \frac{n}{2N_{\text{ph}}}\right), \end{array}$$

where *n* = 1, or *n* = any nonzero odd integer less than *N*_ph_, such that *n* and *N*_ph_ do not share any common factors. It should be noted that a good design necessitates a high coupling between the magnets and the coils, which implies that *n*/*N*_ph_ should be no greater than about 0.6. For a PMV machine with FSCW, a large number of slots will result in a small per slot cross-section area and a large number of PMs which further reduce utilization of PMs and improve the operating frequency. Hence, *S* cannot be too large. However, to improve the reliability further, electrical machines are generally designed with a high phase number, where a corresponding increase in the number of slots happens according to (5). As a result, a compromise between *S* and *N*_ph_ is obtained with a selection of *S* = 20 and *N*_ph_ = 5. Thus, three basic parameters of an “original PM machine” have been determined, in which *S*, *N*_ph_, and *p*_*r*_ are 20, 5, 9, or 11, respectively.

**Figure 1:**
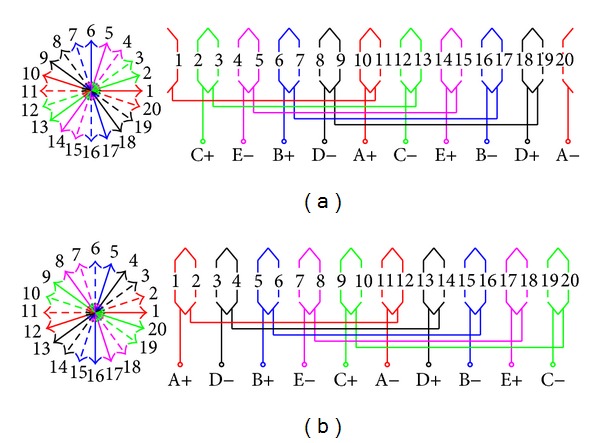
Winding configurations. (a) *p*_*r*_ = 9. (b) *p*_*r*_ = 11.

**Figure 2:**
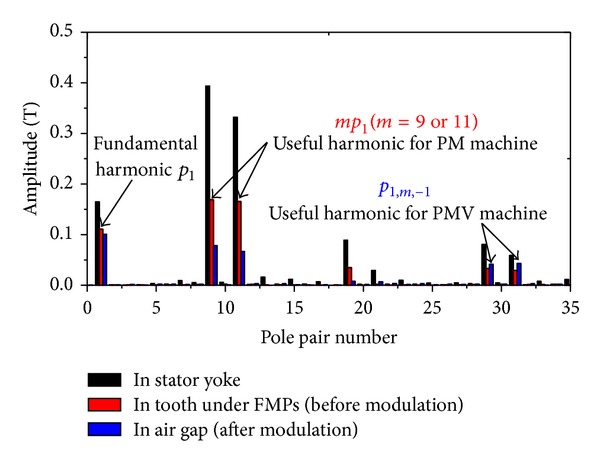
Space harmonic spectrum due to armature windings.

The winding configurations with *p*_*r*_ = 9 and 11 are shown in Figure 1. Clearly, differing from the *p*_*r*_ = 11 winding connection, the *p*_*r*_ = 9 winding connection only has the opposite direction of the rotating magnetic field. It means that they will share the same space harmonic spectrums due to armature windings. So, the space harmonic spectrum of them is shown in Figure 2. It can be seen that the order of the biggest space harmonic content *mp*_1_ is 9 or 11 before modulation, but the fundamental stator space harmonic *p*_1_ is 1th. Obviously, *mp*_1_ = *p*_*r*_ and *m* ≠ 1 due to this kind of SL-FSCW configuration, validating the theoretical analysis in Section 2.

For the stator structure, only one adjustment is needed to transform the “original PM topology” to its corresponding PMV topology. It is to split the stator tooth into *n*_*s*_ FMPs. By considering manufacturing technology, symmetry, vernier effect, and electromagnetic performance, *n*_*s*_ = 40 is a better choice [15]. Up to here, basic structures of two prototypes have been designed as listed in Table 2. From Figure 2, it can be clearly seen that the amplitudes of most harmonics are reduced in the air gap since the different permeances exist in the full flux path from stator yoke to air gap. However, the increased amplitudes of 31st and 29th are significant, which can be known from the comparison of red and blue columns. The reason is that the increased parts of 31st and 29th result from the maximum magnetic field components (9th and 11th) by modulation effect, which is in agreement with the theoretical analysis in Section 2. Thus, in order to produce the maximum torque, the number of PM pole pairs *p*_2_ should be equal to 31 or 29 as listed in Table 2.

**Figure 3:**
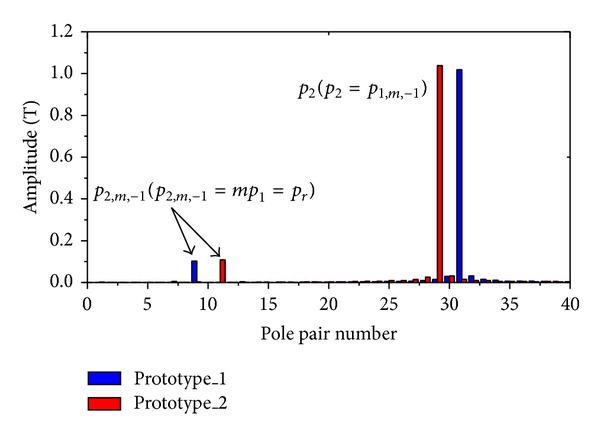
Space harmonic spectrum due to PMs.

Furthermore, Figure 3 compares the space harmonic spectrums of both prototypes in the air gap due to the PMs. It also can be seen that, corresponding to *p*_2_ pole pairs of the rotor rotating field, the highest asynchronous harmonic component is that with *p*_2,*m*,−1_ pole pairs (*p*_2,*m*,−1_ = *mp*_1_). Obviously, the effect of flux modulation is verified by Figures 2 and 3. A fault-tolerant machine with large number of PM poles can be designed by introducing the vernier structure, rather than the increase of the volume and the number of slots.

**Figure 4:**
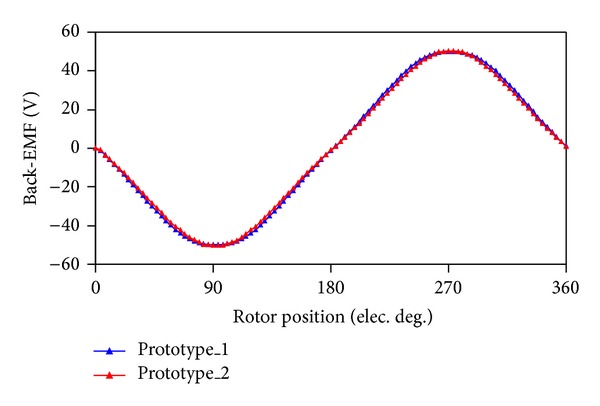
Comparison of back-EMF.

**Figure 5:**
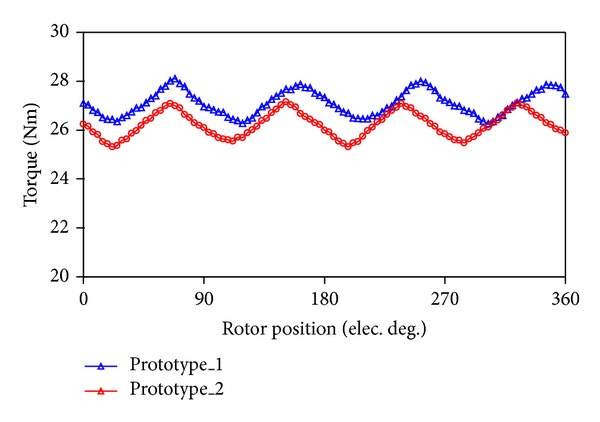
Comparison of output torque.

In order to find which prototype possesses the advantageous performance, Figure 4 shows the back-EMF waveforms at the speed of 600 rpm, and Figure 5 shows the output torque waveforms under the rated operation. It can be known that back-EMFs of both machines are almost the same, but that of the prototype_1 is a little more sinusoidal, and the root mean square (RMS) value is 17.1 V. As a result, the average output torque as shown in Figure 5 of prototype_1 is 1 Nm larger than that of prototype_2. Finally, prototype_1 is selected.

### 3.3. Optimization of Air-Gap Length

**Figure 6:**
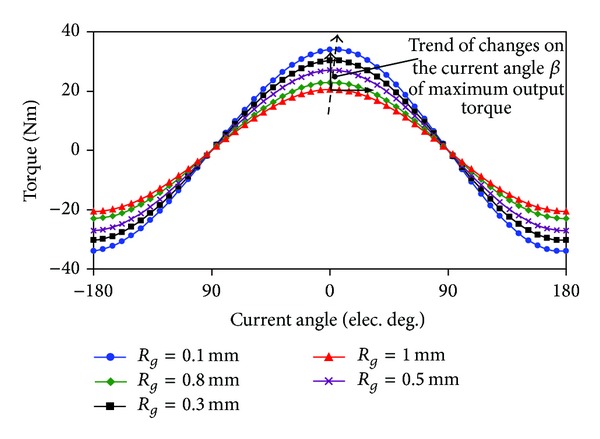
Variations of torque characteristic with air-gap length *R*_*g*_.

As discussed above, PMV machines are very sensitive to the air-gap length.  Figure 6 shows the variation of torque characteristics with air-gap length *R*_*g*_. The torque transmission characteristics are obtained based on the locked-rotor operation. Obviously, the maximum output torque increases linearly when *R*_*g*_ decreases. In addition, it can be seen that the current angle *β* is almost equal to zero when *R*_*g*_ is large, and *β* increases with the decrease of *R*_*g*_. This phenomenon occurs due to the flux concentration and the saturation at the FMPs top. Meanwhile, it indicates that *L*_*d*_ = *L*_*q*_, and thus reluctance torque has no contribution to the output torque when *R*_*g*_ is large. By considering machining accuracy of the air-gap, *R*_*g*_ = 0.5 mm is chosen. With *R*_*g*_ = 0.5 mm, *L*_*d*_ can be thought to be equal to *L*_*q*_ approximately, which means that the maximum torque per ampere control can be achieved by “*i*_*d*_ = 0” control method.

### 3.4. Magnet Design

**Figure 7:**
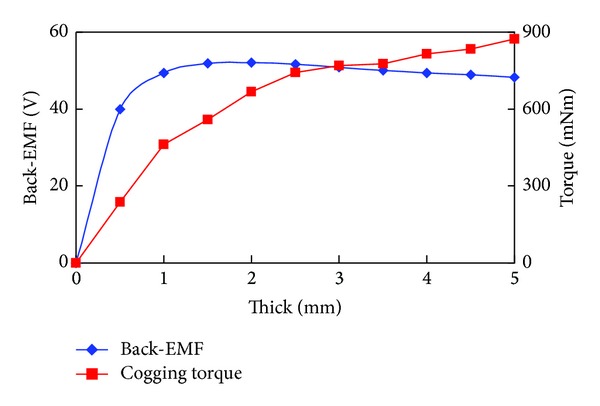
Peak value variations of back-EMF and cogging torque with PM radial thickness *H*_pm_.

The PM radial thickness *H*_pm_ is a significant parameter since it affects the flux density in the air gap, the demagnetization withstand capability, and the cost. In general, the increase of *H*_pm_ can enhance air-gap flux density and thus torque in PM machines. However, for surface-mounted PMV machines, the main function of *H*_pm_ is to increase the resistance to demagnetization. The peak value variations of the back-EMF and the cogging torque with *H*_pm_ are shown in Figure 7. Clearly, the amplitude of cogging torque increases with *H*_pm_ increasing all along, but only when *H*_pm_ < 2 mm, with *H*_pm_ increasing, the amplitude of back-EMF increases. In fact, there is an optimal value of *H*_pm_ corresponding to the largest amplitude of back-EMF. It differs from PM machines where the larger the *H*_pm_ is, the larger the peak value of back-EMF becomes. This is due to the fact that the reluctance is also increased with the increasing *H*_pm_. The permeance of PM is close to air which is far greater than that of stator or rotor iron. The reluctance increase will reduce space harmonic field in air gap, thus reducing the back-EMF amplitude. Hence, the value of *H*_pm_ must be optimized by considering the back-EMF amplitude.

**Figure 8:**
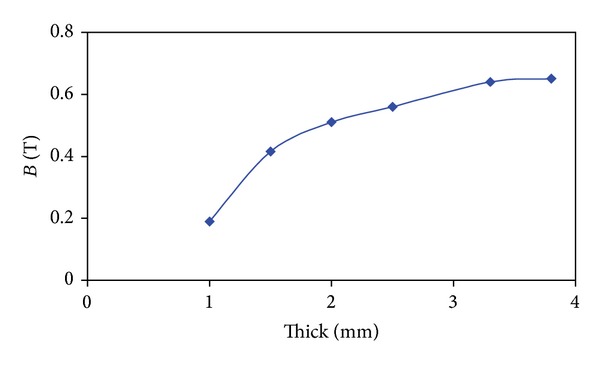
Variation of minimum flux density in PMs with PM radial thickness *H*_pm_.

Partial demagnetization of PMs can influence motor performances of both voltage and torque seriously. The motor temperature, the magnitude of current, and the power angle can determine the partial demagnetization [16]. In this work, the partial demagnetization is investigated when *i*_*d*_ = *i*_*s*_ and *i*_*q*_ = 0 strategy is adopted at the operation temperature of 120°C. It means that the rated current is completely used for demagnetization in order to simulate an abominable operating condition for PMs. By this way, the variation of the minimum flux density in PMs with PM radial thickness is shown in Figure 8. It can be seen that the increase of  *H*_pm_ can effectively increase the resistance to demagnetization. As NdFe35 PM material is usually employed, the flux density in PMs less than 0.2 T can be thought as irreversible demagnetization. As a result, *H*_pm_ = 3.2 mm is selected. With *H*_pm_ = 3.2 mm, the proposed machine can provide a good back-EMF. Moreover, it can avoid the irreversible demagnetization of the PMs to a great extent.

**Figure 9:**
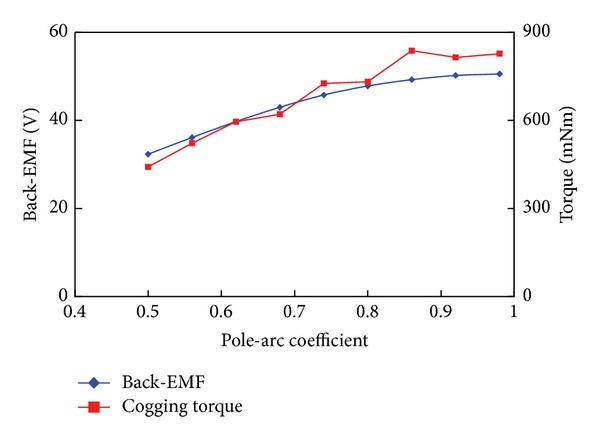
Peak value variations of back-EMF and cogging torque with PM pole-arc coefficient *α*.

The pole-arc coefficient *α* of PMs also has great effects on back-EMF and cogging torque. An appropriate *α* can increase the back-EMF and reduce the cogging torque. In most cases, a compromise between the low cogging torque and high back-EMF should be considered. For a PMV machine, its *α* only influences the peak values of the back-EMF and the cogging torque, rather than their shapes.  Figure 9 shows the peak value variations of the back-EMF and the cogging torque with different pole-arc coefficient *α*. It can be seen that the peak cogging torque is relatively small, although it increases when *α* increases. However, the peak back-EMF increases greatly when *α* increases. Finally, by considering the easy assembling, *α* = 1 is selected.

### 3.5. Optimization of Tooth Width

**Figure 10:**
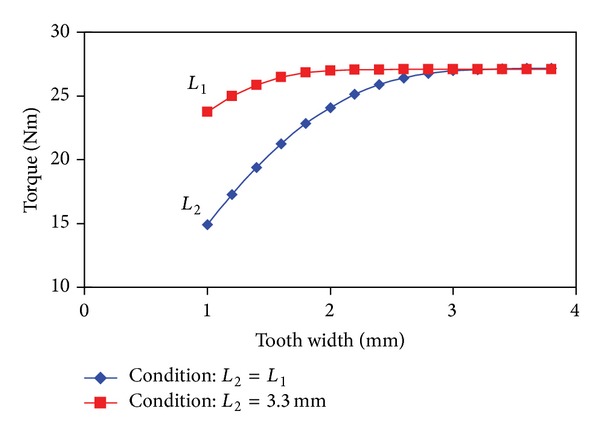
Variation of average output torque with fault-tolerant tooth width *L*_1_ and armature tooth width *L*_2_.

Tooth width can affect slot area and saturation level on tooth; thus it has a great influence on output torque. With SL-FSCWs, the proposed FT-PMV machine has a coil wound on alternate teeth called armature tooth. The unwound teeth are usually called fault-tolerant tooth, which provide a special flux path to decrease the electromagnetic coupling between phases. Tooth width of fault-tolerant tooth *L*_1_ can be unequal to that of armature tooth *L*_2_ to optimize the flux linkage and enlarge the slot area as possible without performance degradation [13]. The variation of average output torque with tooth width is shown in Figure 10. Firstly, *L*_2_ can be optimized by assuming *L*_2_ = *L*_1_ as shown in Figure 10 (blue curve). It can be seen that the average output torque tended to be a constant when *L*_2_ and *L*_1_ are over 3.3 mm. Then, after determining *L*_2_ = 3.3 mm, the optimal value of *L*_1_ can be found as shown in Figure 10 (red curve). Clearly, the average output torque remains unchanged when *L*_1_ > 1.9 mm, which means that all the flux lines can run through the fault-tolerant tooth just with *L*_1_ = 1.9 mm. Finally, *L*_2_ = 3.3 mm and *L*_1_ = 1.9 mm are selected, which have the same effect as *L*_2_ = *L*_1_ = 3.3 mm. Based on this optimal value of tooth width, the slot area can be significantly enlarged.

### 3.6. Final Solution

**Figure 11:**
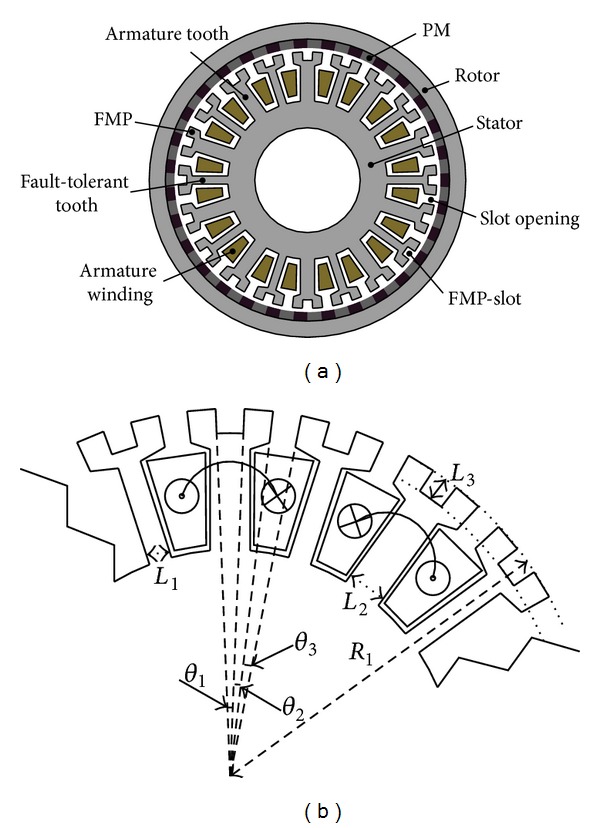
Proposed FT-PMV machine. (a) Cross section. (b) Parameters.

The detailed dimensions, materials, and structure parameters are listed in Table 3. The cross section of the proposed FT-PMV machine is shown in Figure 11(a). Meanwhile, a partial amplification of the cross section is given in Figure 11(b), in which the corresponding important structure parameters are marked. It should be noted that, in this design, the FMP-slot width *θ*_1_, FMP width *θ*_2_, and stator slot opening width *θ*_3_ are made equal (*θ*_1_ = *θ*_2_ = *θ*_3_). In addition, FMP-slot depth *L*_3_ is set to be 4 mm. In fact, as *L*_3_ changes between 2 mm and 5 mm, the performances of the proposed machine almost remain unchanged.

## 4. Simulated Performance

In order to verify the advantages of the proposed FT-PMV machine, its performance is analyzed by using TS-FEM.

### 4.1. Torque Performance

**Figure 12:**
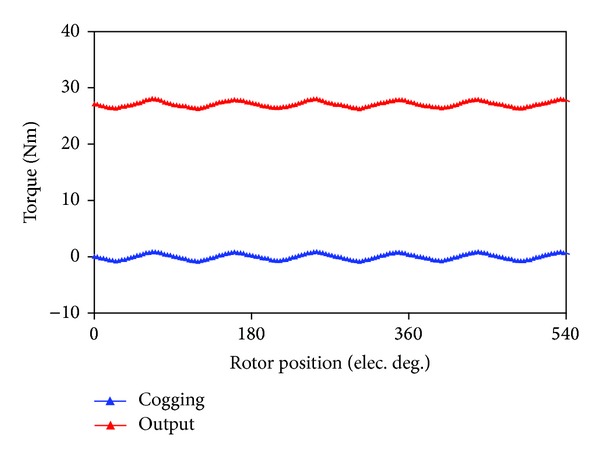
Cogging torque and output torque waveforms.

While the FMPs provide unique magnetic path to modulate between the rotor field and the stator field, there are different air-gap lengths at different positions, for example, different magnetic resistance. Thus, the interaction between the FMPs and the PMs causes the cogging torque.  Figure 12 shows the predicted cogging torque. The peak value of the cogging torque is 0.88 Nm. In addition, the static torque waveform of the proposed FT-PMV machine in BLAC operation mode is also shown in Figure 12. It can be clearly observed that the output torque ripple induces mainly due to the cogging torque. It has been known that, for the PM machines operated in BLAC mode, the harmonic distortion in the back-EMF and the cogging torque are the two main sources of ripple in the output torque [16]. For a five-phase machine only the 5*k* ± 1th (5*k* ± 1 ≠ 3*j*, *k*, *j* is an integer) harmonic components will contribute to the generation of torque ripple. In fact, the harmonic components of the back-EMF of the proposed machine will hardly ever result in the torque ripple, which will be verified in Section 5.

### 4.2. Fault-Tolerant Capability

The self-inductance and the mutual inductance between phases have been calculated as listed in Table 4. It can be seen that the proposed machine has the very low ratios of the mutual inductance to the self-inductances, showing that there is nearly zero magnetic coupling between phases. Namely, a fault in one phase does not undesirably affect the remaining healthy phases. Besides, inductance affects the power factor greatly, which can be seen from the following formula:

(6) $$\begin{array}{c} \text{PF}=\frac{1}{\sqrt{1+\left(L_{s}I/\psi_{m}\right)}}, \end{array}$$

where *ψ*_*m*_ is the magnet flux linkage, *I* is the rms phase current, and *L*_*s*_ is the synchronous inductance. However, the influence of inductance to the power factor is not absolute. In fact, when the electric load of the motor is determined, the change on power factor would be unconspicuous, regardless of the changes of other parameters.

**Figure 13:**
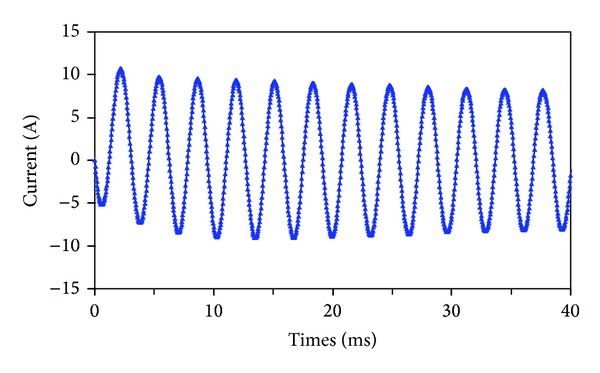
Short-circuit current.

Figure 13 shows the current responses under the short-circuit fault condition at the speed of 600 rpm. Only one phase is short-circuit, and other phases operate normally. It can be seen that the short-circuit current is limited well. Hence, it can be concluded that the proposed machine can offer high fault-tolerant capability.

## 5. Experimental Validation

**Figure 14:**
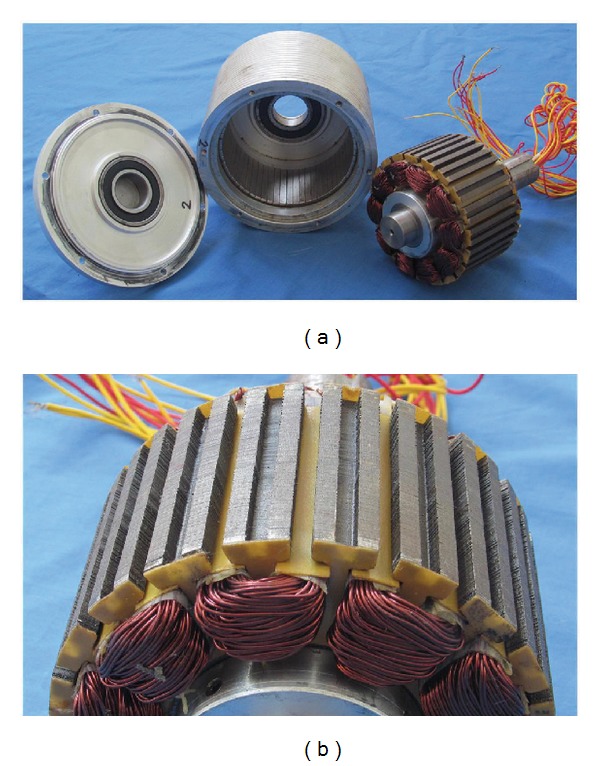
Prototype machine. (a) Stator and rotor assembly. (b) FMPs in stator.

The proposed FT-PMV machine is fabricated and tested to verify the theoretical analysis, which is shown in Figure 14. Clearly, the above discussed fault-tolerant tooth, unequal tooth width, SL-FSCW, and FMP design are adopted in the stators of the prototype.

**Figure 15:**
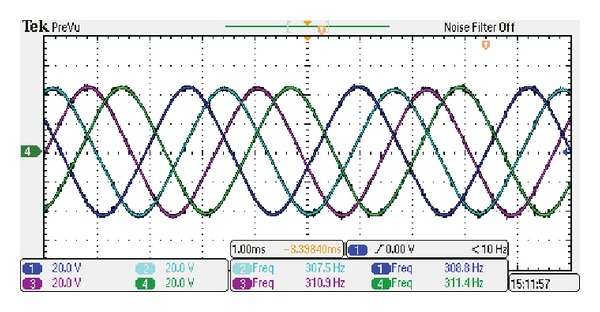
Measured phase back-EMFs (1 ms/div, 20 V/div).

**Figure 16:**
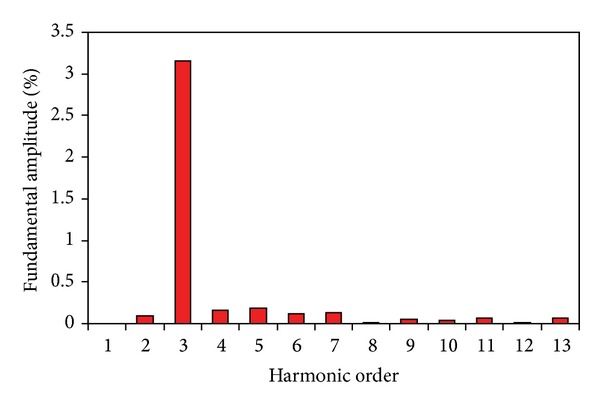
Harmonic distributions of back-EMF.

The measured phase back-EMFs at the speed of 600 rpm are shown in Figure 15, which agrees with the simulated results in Figure 4. The measured value is about 8% lower than the predicted; both lamination factor and end effect are the key affective factors that contributed to this result. Furthermore, the harmonic spectrum of the measured phase back-EMFs is normalized by the respective fundamental components as shown in Figure 16. The total harmonic distortion (THD) is 3.2%. Hence, the proposed machine is very suitable for BLAC operation. Moreover, it can be seen that the contents of harmonics of the proposed machine which is useful for the torque ripple are almost none. Hence, the output torque ripple mainly results from the cogging torque.

**Figure 17:**
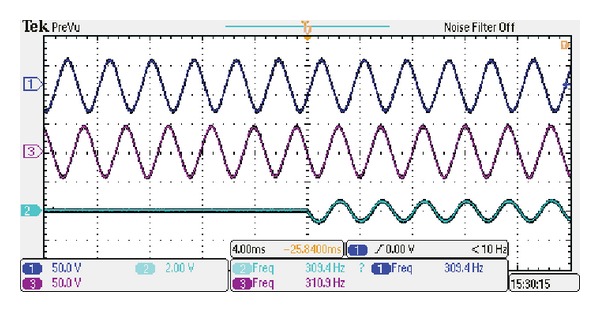
Measured short-circuit current and adjacent phase back-EMFs (4 ms/div, 50 V/div, 20 A/div, 50 V/div).

Figure 17 shows the measured current responses under the short-circuit fault condition at the speed of 600 rpm. Also, the measured adjacent phase back-EMFs are shown together. It can be seen that the short-circuit current is limited well, which agrees with the simulated results in Figure 13. Meanwhile, it can be observed that the back-EMFs are not affected entirely by the adjacent short-circuit current. So, it can be concluded that the independence between adjacent phases in the proposed FT-PMV machine is high, and it can offer high fault-tolerance [16].

## 6. Conclusion

In this paper, a FT-PMV machine has been designed, analyzed, and tested. The operation principle of the proposed machine has been presented. The design process and optimization have been discussed specifically, such as the combination of slots and poles, the winding distribution, and the dimensions of PMs and tooth width. By using the TS-FEM, the machine performance has been assessed. Compared with its PMV counterpart, the proposed machine can offer high fault-tolerant performance, thus improving the machine reliability. Compared with its fault-tolerant counterpart, the PMV structure of the proposed machine can offer a high torque density nearly up to 30 Nm/L, thus reducing the machine volume and weight. Finally, a prototype has been manufactured, and experimental results are provided to validate the theoretical analysis.

## Figures and Tables

**Table 1:** Machine design requirements.

Rated output power, *P*	1.8 Kw
Rated current, *I*_*s*_ (RMS)	10 A
Rated speed, *n*	600 rpm
Cooling method	Natural air convection

**Table 2:** Basic parameters of two prototypes.

Items	*S*	*p*_*r*_	*m*	*p*_1_	*n*_*s*_	*p*_2_(*n*_*s*_ − *mp*_1_)
Prototype_1	20	9	9	1	40	31
Prototype_2	20	11	11	1	40	29

**Table 3:** Machine design requirements.

Items	FT-PMV machine
Phases, *N*_ph_	5
Number of stator slots, *S*	20
Number of rotor pole pairs, *p*_2_	31
Number of FMPs, *n*_*s*_	40
Gear ratio, *G*_*r*_	−31 : 9
Rated voltage, *U* (V)	70
Current density, *J*_*a*_ (A/mm^2^)	6.57
Outside stator radius, *R*_1_ (mm)	60
Outside rotor radius, *R*_2_ (mm)	70
Stack length, *L*_*s*_ (mm)	60
Air-gap length *R*_*g*_ (mm)	0.5
PM thickness, *H*_pm_ (mm)	3.2
Fault-tolerant tooth, *L*_1_ (mm)	1.9
Armature tooth, *L*_2_ (mm)	3.3
PM pole-arc coefficient, *α*	1
Per slot area, *A*_slot_ (mm^2^)	121.8
Slot packing factor, *k*_*s*_	0.6
Stator turns per coil, *N*_*c*_	48
PM type	NdFe35

**Table 4:** Comparison of inductances.

Items	A-A	A-B	A-C	A-D	A-E
Inductance (mH)	3.22	0.0033	0.0113	0.0113	0.0033
Ratio (%)	100	0.102	0.35	0.35	0.102

## Nomenclature

*α*: Pole-arc coefficient
*H*_pm_: Permanent magnet thickness
*τ*: Pole pitch
*N*_ph_: Number of phases
*A*_slot_: Per slot area
*J*_*a*_: Current density
*N*_*c*_: Stator turns per coil
*R*_*g*_: Air-gap length
*L*_1_: Fault-tolerant tooth width
*L*_2_: Armature tooth width
*L*_3_: FMP-slot depth
*L*_*s*_: Stack length
*R*_1_: Outside stator radius
*R*_2_: Outside rotor radius
*θ*_1_: FMP-slot width
*θ*_2_: FMP width
*θ*_3_: Stator slot opening width
*p*_1_: Pole pairs of the fundamental stator space harmonic
*p*_2_: Pole pairs of the PMs of PMV machines
*p*_*r*_: Pole pairs of the PMs of conventional PM machines.
